# Supplementary material for: Are carbon emissions trading and green financial instruments synergistic? -Comprehensive quantitative research based on content analysis
Source: PLoS One. 2024 Mar 7;19(3):e0298601. doi: 10.1371/journal.pone.0298601 (PMC10919601; doi:10.1371/journal.pone.0298601)
Supplement: S1 Data — (ZIP) [file pone.0298601.s001.zip › Data/地级市Z_F工作报告.zip!/░▓╗╒/╗┤▒▒╩╨/└┤╫╘╣1⁄2╓┌║┼í╛┬φ┐╦╩2╛▌═°í┐_2009.docx]

各位代表:

    现在,我代表市人民政府,向大会报告工作,请予审议,并请市政协各位委员和其他列席人员提出意见。

    一、2008年主要工作回顾

    刚刚过去的一年,是我市改革发展进程中非常重要也极不平凡的一年。一年来,在市委的坚强领导下,在市人大、市政协的监督支持下,市政府团结和带领全市人民,认真贯彻党的十七大、十七届三中全会和胡锦涛总书记视察安徽重要讲话精神,深入学习实践科学发展观,牢牢把握机遇,积极应对挑战,圆满完成了市十四届人大一次会议确定的目标任务。预计全年实现生产总值350亿元,比上年增长14.5%;规模以上工业企业完成增加值183亿元,增长20.1%;完成固定资产投资204亿元,增长35%;社会消费品零售总额88.7亿元,增长22.5%;财政收入43亿元,增长20.6%;城镇居民人均可支配收入 12850元,增长10.4%;农民人均纯收入4100元,增长21.5%,综合经济实力迈上了一个新台阶。

    (一)工业经济提速增效,规模企业迅速增加,工业主导地位持续提升。大力实施“3588”工业振兴计划,扎实开展“工业项目推进年”活动,跟踪帮扶重点困难企业,多措并举推动工业经济快速增长。预计全市规模以上工业增加值增幅提高4.9个百分点,拉动全市经济增长9.8个百分点;经济效益综合指数创历史最高水平,工业产品销售率居全省第一。大力优化工业经济结构。在做大做强煤电、煤化工产业的同时,积极推进山河智能、思朗食品、华孚色纺等重大项目建设,煤化工和非煤产业比重持续提升,六大主导产业基础逐步形成。积极培育壮大市场主体,全年新增规模以上工业企业超过150家。加快推进企业退城进区,11家企业进入开发区和工业项目区。大力推进技术创新和技术进步。组织实施技术改造项目168个,完成工业技改投资70亿元,增长25.5%;竣工项目78个,年可新增工业增加值8亿元。中润公司生物化工及能源转化技术取得重大突破。淮北矿业集团企业技术中心、中煤特凿公司煤矿深井建设技术工程实验室进入国家级行列,实现全市零的突破。500千伏淮宿变电站工程获2008年度中国建筑工程“鲁班奖”。大力发展循环经济,狠抓企业节能降耗。组织实施了临涣焦化一期、烈山区洪庄村循环经济工程和国安电力能量系统优化、维科印染余热回收利用等节能项目,争取中央和省财政预算补助7853万元。预计万元生产总值能耗下降4.5%,工业固体废物综合利用率达93%以上。

    (二)农业生产稳定发展,产业结构继续优化,新农村建设扎实推进。认真落实强农惠农政策,继续实施小麦高产攻关,夏秋两季双获丰收,粮食总产连续三年创历史新高。植树造林面积完成1.3万亩。养殖业发展迈上新台阶,全年肉蛋奶总产12万吨,增长7.7%;生猪规模养殖比重位居全省前列,9个产品、4个基地获国家无公害农产品认证。农业产业化进程不断加快,全年新增规模以上食品加工企业14家,省级农业产业化龙头企业4家,市级示范性农民专业合作组织26家。投资4.5亿元的宝迪食品工业园一期正式生产,雨润集团3000万只肉鸡加工项目、思朗食品年产4.5万吨饼干项目正在抓紧建设。“万村千乡市场工程”试点工作连续三年获省政府表彰,中瑞农副产品批发市场成为全国100家重点改造的农产品批发市场,凤凰山农副产品批发市场开工建设。新农村建设“五镇十村”示范工程进展顺利。农田水利基本建设、中低产田改造步伐加快,农业生产条件进一步改善。农村劳动力转移就业力度加大,对2.7万人进行了技能培训,转移就业38.1万人。

    (三)生态城市启动建设,城市功能日臻完善,管理水平不断提高。坚持规划引领,开始编制生态城市建设总体规划,完成了南湖、相湖、东湖景区和黄里生态园规划编制,“六湖九河”水景观规划初步审定。加强城市生态环境建设,时代公园、人民路绿化改造和新城区截污引水及环境治理一期、污水处理二期工程完工,东部荒山绿化工程开始实施。积极推进城市东进南扩,编制了东部新城区概念性规划,栗苑路、长山南路建设进展顺利,人民路东扩段开工建设。积极推进老城区改造,淮北火车站改造完成前期准备,城区防洪排涝工程加快建设,长山路沟中段全线贯通。完成天然气利用一期工程,近11万户居民用上天然气。完善住房供应体系,全年房地产开发建设面积330万平方米,完成投资22亿元,分别增长 39%和25%。我市规模最大的塌陷区村庄搬迁工程———仁和小区主体工程完工,将解决6个自然村、1.2万人的住房问题。繁荣发展商贸流通,中泰置业广场二期等10大重点项目取得突破性进展,锦华苑社区被评为全国商业示范社区。加强市容环境综合整治,数字化城管建设获得全省人居环境范例奖,我市以全省第二名的成绩被评为安徽省首届文明城市,创建全国文明城市、全国优秀旅游城市和全国无障碍建设城市工作取得新成效。

    (四)重大项目加快建设,固定资产投资快速增长,发展后劲不断增强。强化市级领导联系项目制度,重点加强对20项重大基本建设项目和20项重大技改项目的跟踪调度,列入省 “861”行动计划的重大项目提前超额完成年度投资计划。煤电项目全面推进,孙疃矿、卧龙湖矿、五沟矿、界沟矿竣工投产,新增核定生产能力390万吨/ 年;杨柳矿、刘店矿、临涣选煤厂扩建、凤凰山输变电工程等进展顺利。临涣煤矸石电厂一期并网发电,大唐淮北虎山发电厂项目前期工作基本完毕。煤化工项目取得新突破,临涣焦化一期项目4台焦炉和焦炉气制甲醇已正常生产,二期获省“三高”项目资金支持;南坪煤化工项目确定了淮北矿业集团和上海焦化的增资扩股方案,去年底完成公司更新注册登记。加工制造业项目加快建设,丰盛泰重工基地、恩远铝业二期工程、富马酸及粗苯加工系列项目建成投产,天地通铸造加工项目扎实推进。基础设施项目建设步伐加快,临涣工业园引水工程主管道贯通,萧淮路改建一期工程实现半幅通车,五宋路改造过半,化家湖水库除险加固主体工程基本完成。抢抓国家扩大内需的政策机遇,迅速谋划上报3批项目。

    (五)招商引资成效显著,重点领域改革深入推进,发展活力日益彰显。主动参与泛长三角发展分工与合作,大力开展产业招商、驻点招商,组团参加徽商大会、中博会、高交会和中国国际投资贸易洽谈会,成功举办了2008中国(淮北)专利技术推介会、煤矿机械投资贸易洽谈会和海外华商投资项目推介会。预计全年引进市外资金150.5亿元人民币,增长21.4%;实际利用外资首次突破1亿美元,增长62.9%;美国高盛、湖南山河智能、香港锦泰食品、江苏雨润、上海索谷等国内外知名企业投资淮北。对外贸易规模扩大,进出口总额突破1亿美元大关,增长50%。深化投融资体制改革,组建了市建投集团。完善金融机构目标考核奖励办法,加强信用平台建设,做大做强贷款担保机构,组织开展了4次银企对接活动,预计全年各项贷款余额较年初增加30亿元。加快利用资本市场步伐,重点培育了10家上市资源企业。继续推进各项改革,淮北矿业集团完成主辅分离, 淮海集团公司挂牌运行。53家经营服务性事业单位完成改制任务。进一步清理行政审批项目和行政事业收费,年可减轻企业和社会负担2200多万元。积极稳妥开展农村土地承包经营权流转改革试点,新增土地流转面积3万亩。建立财政预算编制、执行、监督“三权分离”机制,预算编制进一步规范化、科学化和制度化。大力发展非公有制经济,积极推进全民创业,全年新增个体工商户6100户、私营企业720家、从业人员2万人,凤凰山工业项目区被列为省创业基地和全国农产品加工创业基地,我市被评为全省发展非公有制经济先进市。

    (六)园区建设步伐加快,县区经济高速增长,拉动作用更加突出。设立园区发展专项资金,在政策、项目用地等方面支持开发区和工业园区建设,园区产业集聚效应和辐射带动作用明显提高。全市园区建成区面积39.5平方公里,完成固定资产投资61.5亿元,增长73.2%;新引进外来投资项目110个,实际利用市外资金62.3亿元。市经济开发区实现经营收入38亿元,增长31%。统筹县区经济社会发展,进一步调动县区发展的积极性,制定出台了《关于进一步支持县区加快发展的意见》,扩大县区经济管理、城市建设管理、社会管理、公共服务、人事和机构管理等5个方面29项管理权限,县区经济呈现速度加快、活力增强、各具特色、竞相发展的良好态势。去年,县区完成生产总值155亿元,增长 18.9%;固定资产投资108亿元,增长72.8%;规模以上工业增加值42.5亿元,增长60.9%,对全市经济增长的贡献率达到54.5%。

    (七)民生工程扎实推进,社会建设得到全面加强,和谐淮北加速构建。各级财政共投入资金4.7亿元,实施了18项民生工程,让改革发展的成果覆盖千家万户、惠及百姓人家。加强就业和社会保障工作。全面落实就业再就业优惠政策,积极开发就业岗位,全市城镇新增就业3.3万人。加大社会保险扩面力度,基本养老、失业、医疗、工伤保险覆盖率达95%以上。全面提高企业退休人员养老金,4.53万名退休人员人均月增养老金97元。新被征地农民基本养老保险,实现即征即保。完善农村居民最低生活保障制度,受益农民3.9万人。提高农村“五保户”供养标准,近万人享受五保供养政策。建立城镇未参保集体企业退休人员基本生活保障制度,920人享受基本生活补助。积极开展救灾救济救助工作,发放雪灾、低温冷冻等自然灾害和“两节”送温暖活动救助资金1250万元。加快经济适用房建设,总面积13.3万平方米的南黎花园交付使用,解决了1092户低收入家庭住房困难;滨河花园一期工程楼栋全部封顶;淮北矿业集团完成棚户区改造5.2万平方米。城乡医疗健康工程扎实推进。实施城镇居民医疗保险制度,全市参保人数达到44万人。调整城镇职工和城镇居民基本医疗保险报销比例和最高支付限额,医疗保险待遇水平大幅提高。新型农村合作医疗参合率达到97.4%,高出全省平均水平6个百分点;建设社区卫生服务机构100所,建设规范化乡镇卫生院和村卫生室102所。组织弱视儿童义诊,实施白内障免费手术1000例。农村饮水安全工程加快推进,10多万人安全饮水问题得到解决;广播电视 “村村通”工程进展顺利,农村公路“村村通”工程完成212.5公里。城乡义务教育经费保障机制改革全面实施。免除了义务教育阶段30万名城乡学生学杂费、20万名农村学生教科书费,2892名贫困寄宿学生得到生活补助,1.5万名高校和中等职业学校困难学生得到资助。改造农村中小学D级危房2.2万平方米;投入资金1亿元,有效化解农村义务教育债务。

    社会事业全面发展。高度重视科技工作,首次设立了科学技术研究与开发重大专项;精细化工专业型科技企业孵化器挂牌运行,实现了孵化器建设零的突破;濉溪县被列入国家科技富民强县试点县,五沟镇、濉溪镇、段园镇被列入省科技创新试点镇。大力发展教育事业,建立了全市教师补充与优化长效机制,普通中小学教育质量稳步提升,高考再创佳绩,职业教育、成人教育、民办教育、特殊教育取得新成绩;优化主城区教育布局,实施义务教育集团化办学试验,新建住宅小区配建义务教育阶段学校 3所。加快发展文化事业,进一步丰富群众文化生活,电视剧《大嫂》在央视热播,第三次全国文物普查有序推进。积极开展全民健身运动,实施农民体育健身工程,成功承办了全省第二届特奥运动会。认真落实农村计划生育家庭奖励扶助制度,农村计生工作不断加强;城市流动人口计生工作全省领先,相山区被评为国家计划生育住处化管理先进集体。大力加强环境保护和节能减排工作,二氧化硫排放量下降5.9%,化学需氧量下降11.3%。积极推动土地集约节约利用,开展了闲置土地和擅自改变土地用途清理处置工作。抓好价格监控,稳定民生价格。残疾人事业加快发展,综合服务基地建成使用,备战第十三届残奥会工作受省政府表彰。农业普查工作获得全国先进,统计服务水平进一步提高。工商管理、质量监督、食品药品管理、贸易促进、双拥、国防、防震减灾等工作继续加强。外事侨务、民族宗教、审计、人事编制、旅游、机关事务管理、气象、对台、保密、档案、地方志、妇女、儿童、老龄等工作取得新成绩。

    社会大局保持稳定。建立和完善突发公共事件应急预案,成功举行粗苯泄露事故、突发交通事故等应急救援演练。广泛开展爱国卫生运动,有效组织了疟疾休根治疗、手足口病防控。全面加强市场监管,及时筛查救治患儿,妥善处置了食用三聚氰胺奶粉事件。积极排查化解矛盾纠纷,实施信访案件“百案攻坚”,切实做好人民来信来访和行政复议工作。认真落实安全生产责任制和责任追究制,加强重点行业和领域专项整治,安全生产形势持续稳定。全力做好奥运会和残奥会安保工作,切实加强社会治安综合治理,深入开展平安淮北建设活动,严厉打击各类违法犯罪活动,全年刑事案件发案数大幅下降,社会秩序保持平稳。援川警队受到省政府表彰。

    (八)行政效能不断提升,民主法制积极推进,政府自身建设进一步加强。深入开展“创新促发展、执行看落实”解放思想主题实践活动。进一步完善目标责任制考核办法,主要工作目标实行“一票否决”;加大过程监督和奖惩力度,每季度进行一次动态考核,强化重点工作经常性调度。市行政服务中心在全省首家建立了行政审批电子监察系统。全市公务办公网建设强力推进,272家单位基本实现网上收发文和公文流转。办理人大代表议案和建议119件、政协委员提案178件, 满意率98 %以上。认真听取民主党派、工商联和无党派代表人士的意见,切实加强与工会、共青团、妇联、科协等群团组织的联系。坚持依法行政,规范行政行为,推行行政执法评议考核,强化行政执法责任追究,建立了重大行政决策合法性审查制度,全年审查重大行政决策15项。积极开展“五五”普法宣传和法律援助,全社会法制观念不断增强。畅通“市长热线”,改进“政风行风热线”,建立“12388”信访举报电话,群众反映和解决问题的渠道进一步拓宽。积极开展反腐倡廉制度建设推进年活动,组织开展民主评议机关活动,廉政建设和反腐败斗争取得新成效。

    在经济形势复杂多变、发展约束性因素不断增多的情况下,取得这样的成绩确实来之不易。这是省委、省政府和市委正确领导的结果,是市人大及其常委会、市政协和各民主党派监督支持的结果,更是全市人民迎难而上、奋力拼搏的结果。在此,我代表市人民政府,向全市广大工人、农民、知识分子、干部和离退休老同志,向各民主党派、各人民团体和社会各界人士,向驻淮解放军指战员、武警官兵和政法干警,向中央和省驻淮单位,向所有关心和支持淮北发展的朋友们,表示衷心的感谢并致以崇高的敬意!

    回顾过去一年,我们也清醒地认识到,在前进的道路上,我们还面临着不少困难和问题。一是受全球金融危机的影响,经济平稳较快增长的压力加大,发展不足、发展不快的问题依然比较突出,持续发展的基础还不稳固。二是经济结构调整任务繁重,企业的发展活力有待进一步激发。三是体制机制不活的问题仍较突出,城乡和区域发展还不平衡, 社会事业发展相对滞后,影响社会稳定的矛盾依然存在。四是政府自身建设仍需不断加强,领导科学发展的能力有待进一步提高,投资环境仍需不断改善。这些都需要我们在今后的工作中高度重视,采取更加有力的措施切实加以解决。

    二、2009年政府工作的总体要求和主要预期目标

    今年是我市深入学习实践科学发展观、促进经济社会长期又好又快发展的关键之年。千方百计保持来之不易的良好发展势头,是我们面临的最大挑战,也是最核心的任务。去年以来,次贷危机引发的金融危机愈演愈烈,迅速从局部发展到全球,从发达国家传导到新兴市场国家和发展中国家,从金融领域扩散到实体经济领域,酿成了一场历史罕见、冲击力极强、波及范围很广的国际金融危机。目前,这场金融危机不仅本身尚未见底,而且对实体经济的影响正进一步加深,其严重后果还会进一步显现。受此影响,我国经济增速下滑已成为经济运行中的突出矛盾,对我市的现实冲击也越来越明显,煤、电等主导产业已经受到不同程度的影响。战胜国际金融危机严重冲击,应对前所未有的挑战,需要狭路相逢勇者胜的坚定信念,需要愈是艰难愈向前的拼搏精神,需要咬定发展不放松的顽强意志,要努力从变化的形势中捕捉和把握难得的发展机遇,在逆境中发现和培育有利因素,知难而进,化危为机,加快发展。

    2009年政府工作的总体要求是:全面贯彻党的十七大、十七届三中全会和中央经济工作会议精神,以邓小平理论和“三个代表”重要思想为指导,深入贯彻落实科学发展观,强力推进工业化、城市化和城乡一体化,统筹深化改革与全面开放,统筹扩大内需与拓展外部市场,统筹发展县区和园区经济,统筹经济社会协调发展,保增长、扩内需、调结构,推动经济社会又好又快发展,提高广大人民群众的生活质量和幸福指数。

    综合各种因素考虑,今年经济社会发展的主要预期目标是:地区生产总值增长11%,财政收入增长12%,规模以上工业增加值增长12%,固定资产投资增长25%,社会消费品零售总额增长16%,外贸进出口总额增长10%,城镇居民人均可支配收入增长6%,农民人均纯收入增长8%,城镇登记失业率控制在4.2%以内,万元生产总值能耗下降4.5%,化学需氧量、二氧化硫排放量分别减少1%和 12.16%,人口增长控制在省下达指标以内。

    实现上述目标,做好今年的政府工作,关键是要坚定加快发展、科学发展的信心和决心。困难面前,信心和决心比黄金和货币更重要。“事不避难,知难不难”。从宏观支撑因素来看,国家出台了4万亿元的投资计划,加强道路等基础设施建设,有利于扩大市场对煤炭、煤化工、建材等产品的需求,促进工业的即期增长。国家大幅度提高粮食最低收购价格,新增中央投资主要用于加快民生工程和农村基础设施建设,将进一步增加农民收入，改善农村生产生活条件。国家实施积极的财政政策和适度宽松的货币政策,有利于我市大力调整经济结构,推动虎山电厂、青东煤矿等重大工业项目,以及火车站改造、滨河花园等城市建设项目的实施,也为平山电厂等一批前期重大项目上马带来新机遇。从市域支撑因素来看,经过多年的建设与发展,全市经济抵御风险的能力不断增强。随着一批重大项目陆续投产,全市经济结构将得到进一步优化。随着规模企业的大幅增加,县区经济将继续保持强劲发展势头。随着开发区和工业园区基础设施进一步完善,招商引资和园区经济将会有一个比较大的发展。随着城乡居民收入继续增加,社会保障体系不断完善,重点商贸项目建设, 以及物流等新兴产业的发展,城乡消费市场将进一步活跃。特别是经历了2003年和2008年两次大的宏观调控,我们已拥有结构调整、城市转型和统筹城乡发展等方面的经验和教训,驾驭经济发展的能力明显提高。只要我们进一步坚定发展的信心和决心,积极抓住各种机遇,用足用活各项政策,就一定能够实现各项预期发展目标。

    三、奋力推动经济平稳较快发展

    保持经济平稳较快增长是应对经济危机的基础,是我们改善民生、推动各项社会事业发展的前提,也是上级党委、政府和全市人民的期望。我们将密切关注经济发展走势,努力保持全市经济发展的良好势头。

    (一)抢抓机遇扩大有效投入,加大项目建设力度,培育壮大新的经济增长点。扩大投入规模,既能有效地拉动即期经济增长,又能为长远发展增添后劲;优化投资结构,既有利于优化经济结构,也有利于促进发展方式的转变。越是在困难的时候,越是要紧盯工业项目、狠抓重点项目,坚定不移抓住投入这个牛鼻子不放松。切实加快刘店矿、杨柳矿、临涣选煤厂扩建工程、临涣煤焦化焦炭项目、索谷电缆、思朗食品等重大生产性项目建设,力争早日投产。完成五宋路、萧淮路一期续建, 开工建设泗许高速公路淮北段和泗永路改造工程。落实与省电力公司战略合作协议,加快城乡电网建设。抓紧落实项目开工条件,争取袁店一矿和二矿等项目尽快开工建设。抓住国家和省加快项目审批的有利时机,把项目前期工作做深、做细、做实,积极推进南坪煤化工、国安电力二期、赵集矿、萧淮路二期工程等重大项目前期工作。加强对市场自主投资的鼓励和引导,落实重大项目建设资金。着力抓好项目谋划设计,重点围绕“四加快一提高”的投资导向,在交通、城乡基础设施、节能减排、结构调整、支持“三农”、改善民生等方面,编报一批打基础、管长远、增后劲的大项目。强化市级领导联系项目制度,落实重点项目协调服务责任制,建立统一领导、上下联动、务实高效的项目推进工作机制。

    (二)奋力保持工业经济较快增长,做大做强主导产业,加快推进工业化进程。坚定不移地实施“3588”工业振兴计划,组织开展“企业规模壮大年”活动。进一步强化工业经济运行调度,充分发挥煤电、煤化工主导产业和大企业的支撑作用。落实好市政府《关于促进经济又好又快发展的若干意见》,重点对61户困难企业进行跟踪帮扶。扶优扶强,支持现有工业企业进一步扩大规模,选择80家左右具有发展潜力的企业进行重点培育,力争年销售收入超20亿元企业达到4家、10亿元企业5家、亿元企业30家。深入推进中小企业成长工程,全年新增规模以上工业企业80家。大力推进企业技术创新和技术改造,重点实施重大技术改造项目20个。调整优化工业结构,加快构筑“三足鼎立”产业格局。加快煤电项目,特别是大唐虎山发电厂等项目建设,积极推进临涣煤焦油加工和粗苯精制项目建设;着力培育壮大非煤产业,发挥口子酒业、宝迪食品、六和勤强等企业的辐射带动作用, 着力打造百亿元食品产业;支持华孚色纺、维科印染、今一服装、中盟制衣等企业发展,力争纺织服装产业稳产增效;突出山河智能等企业的引领作用,提高煤矿机械制造产业层次;加快新中源等项目推进步伐,力争陶瓷建材产业上规模、上水平;继续大力发展电子、医药、新材料、精细化工等新兴产业。认真落实国家循环经济试点市实施方案,选好项目,明确主体,扎实推进循环经济试点工作。

    (三)多渠道增加农民收入,加快农村基础设施建设,推动城乡一体化发展。大胆探索,精心组织,积极推进省级城乡一体化综合配套改革试验区建设,加快构筑城乡一体化新格局。一是加大财政资金向农村倾斜的力度,市财政新增1亿元用于城乡一体化建设。二是以市场为导向,建立从种植、养殖到加工、流通有机联结的产业链条,完善技术服务体系,加快发展农村经济。大力实施优质粮食工程,抓好小麦高产攻关活动、规模养殖小区建设活动、高效经济作物栽培小区建设活动,落实玉米振兴计划。大力推进农业产业化经营,推动鲁王、天宏、谷王等加工企业优化升级,加快思朗食品、雨润肉鸡、宝迪食品和六和勤强二期等项目建设,扶持建设30个规范化农民专业合作社。三是多渠道增加农民收入。全面落实国家粮食最低收购价、粮食补贴、农机补贴和小麦高产攻关奖励等各项强农惠农政策。全力以赴做好返乡农民工就业工作,组织开展技能培训,引导就近就业和创业就业。四是大力加强农村基础设施建设,深入推进城乡基本公共服务均等化。推动教育、卫生、文化、社保等公共服务向农村延伸。加强农田水利基本建设,抓好农业综合开发,改造中低产田,完善濉溪县南部农田林网,做好包浍河等小流域综合治理规划和项目申报工作。进一步加强小城镇特别是建制镇建设。五是制定和完善覆盖城乡的一体化规划体系,有序推动农民向城市、矿区、中心镇和中心村集聚,加快城郊村、“空心村”改造,积极推进村庄整治和宅基地置换,完善中心村基础设施和公共服务配套,重点提高公共服务水平,建立城乡一体化的基础设施体系,改善农村生活环境,切实抓好百善镇、五沟镇、渠沟镇、烈山镇、高岳办事处的试点工作。

    (四)继续推进山水生态城市建设,完善城市功能,不断提升城市化水平。围绕建设“山、水、城”相依的宜居城市,继续完善城市控制性详细规划,科学编制东部新城分区规划、核心区控制性规划、水资源配置规划和生态城市建设规划。大力推进城市东进南扩,建成人民路东扩段、新东外环路、沱河路和翠峰南路、栗苑路,实施中湖景区北部景观工程和相山城区湿地绿化,继续实施东部荒山绿化,推进华家湖水库除险加固等重点水利工程建设和国家级节水型社会建设试点工作,进一步推动相濉、相烈、相杜同城化。推进城市防洪工程和市危险废物处置中心建设,争取开工建设市开发区污水处理厂、垃圾发电厂、中心镇污水处理厂等项目。以淮北火车站综合改造为重点,加快老城区和城中村改造。继续推进房地产业稳定健康发展,调整房屋供应结构,逐步形成高中档商品房、经济适用房和廉租住房比例较为合理的住房供应体系。提高住房公积金最高贷款额度、降低首付比例,稳定发展住房消费。充分发挥城市管理监控中心作用,健全城市长效管理机制,深化城市管理相对集中行政处罚权改革,统筹城乡创建协调发展,进一步巩固文明城市创建成果。

    (五)积极参与泛长三角分工合作, 提高招商引资质量,在借力借势发展上寻求新突破。把握国际国内经济结构调整和产业梯度转移新动向,建立政府为引导、市场为主导、企业为主体、项目为载体的协作机制,着力构建市场化、专业化、网络化招商平台,积极引进大企业、大项目。办好2009中国(淮北)专利技术推介会和煤炭机械投资贸易洽谈会。认真贯彻落实市委、市政府《关于积极参与泛长三角区域分工与合作的意见》，重点抓好六大主导产业,加快推进沪皖合作项目———平山电厂申报与建设,积极引进长三角地区的资本、技术与人才,着力打造煤化工产业、纺织产业、机械制造产业集群,建设食品工业高地和陶瓷建材基地。落实好招商引资优惠政策,搞好全程代理服务,建立考核奖惩新机制。全年利用内资187.5亿元、外资1.25亿美元,均增长25%。多渠道筹集建设资金,加快园区科创中心、道路管网和标准化厂房建设,完善功能配套,提高承载能力。落实市经济开发区封闭运行实施办法,加大园区全程代理服务力度,推动园区由单一“管理型”向综合“服务型”转变。明确园区功能定位,引导关联项目向特色园区集中。全年开发区和工业园区完成投资78.5亿元,增长27%。

    (六)大力发展商贸流通,繁荣发展第三产业,扩大消费需求。围绕强化经济增长的支撑力和提高人民群众的生活质量,适应产业结构调整和消费结构升级,促进服务业加快发展。加快启动农村市场。以“万村千乡市场工程”、“双百市场工程”、“新网工程”、“家电下乡工程”为抓手,大力推进农村现代市场体系建设和农产品冷链系统与物流配送、质量安全可追溯系统等流通基础设施建设,着力提高农产品流通现代化水平。积极扩大城市消费。以“社区商业双进工程”、“惠民放心早餐工程”和创建省级“商业示范社区”为抓手,大力推进生活服务性商业网点建设,完善城市消费功能。以“商业节能降耗工程”、“商务诚信建设工程”为抓手,大力推进商贸行业信用体系建设, 倡导绿色消费、安全消费、诚信消费,改革市场管理体制,规范市场秩序,营造良好的商业环境。加快重点商贸项目建设。在继续抓好去年10大重点商贸项目建设的基础上,再筛选出一批商贸项目重点加以推进,带动新老城区购物中心、连锁超市、精品商厦、星级酒店和品牌大型餐饮业等新型业态的发展。大力发展现代物流业。加快建设烈山粮食工业园、百善百路物流中心、相山凤凰山农副产品批发市场、淮北润恒等物流中心,支持中瑞等大型农产品市场继续做大做强。

    (七)加大改革创新力度,发展壮大非公经济,加快释放发展活力。积极发挥银行业金融机构信贷主渠道作用,支持、引导银行业金融机构加大信贷投放。创新政银企合作方式,加大中小企业、重点项目推介和对接力度。扩大市中小企业贷款风险补偿基金和政府性担保资金规模,支持、引导民间资金参与担保体系建设。鼓励金融产品和服务创新。对暂时经营困难的企业,通过贷款展期、债务重组、提供临时性解困资金等方式予以帮助;采取贸易融资、票据贴现等方式为企业提供流动资金。加强上市企业培育,加快利用资本市场步伐。稳步推进小额贷款公司试点工作,引进股份制商业银行,尝试建立工业园区特色支行。支持各类金融组织向农村延伸机构和服务网点,大力推广农户小额信用贷款和联保贷款,建立健全政策性农业保险制度。着力优化金融生态环境。深入推进农村综合改革,在稳定农村基本经营制度的基础上,继续抓好农村土地承包经营权流转,完善集体林权配套改革,探索城乡户籍制度改革,积极推进为民服务全程代理工作。深化事业单位改革,全面完成经营服务性事业单位转企改制任务。积极推进机关后勤管理事业改革与发展。加强国有资产的监管,理顺监管体制。完善和落实发展非公有制经济的政策措施,支持引导民间资本广泛参与民生工程、基础设施和生态环境建设。深入开展全民创业活动,继续加强创业基地和公共服务平台建设,鼓励非公有制企业参与大中型企业产业链配套。加大对通信企业基础设施建设的支持力度,全面提升现代信息技术在公共服务、社会管理和市场监管方面的应用水平。

    (八)优化财政收支结构,充分发挥财政职能作用,促进经济平稳较快发展。进一步加快培植税源,继续加大重点项目建设投入,增加中小企业技术改造、产品研发、生态保护和节能减排等方面的资金投入,落实好开发区和工业园区财税扶持政策。认真落实增值税转型等支持经济发展的财税政策,发挥财政贴息、担保等资金的引导作用,激活更多的社会资本。把握税收政策变动趋势,及时做好收入的预测和分析,努力把税收政策的含金量转化为现实的财政收入。严格依法征税,规范非税收入管理,确保财政收入稳定增长。积极争取国家和省财力支持,争取外国政府和国际金融组织贷款项目。继续调整优化财政支出结构,严格控制一般性支出,强化资金调度,集中财力支持经济发展、城乡建设、民生工程、社会事业等重点领域。加强对行政事业单位公务用车和办公用房等国有资产的监督管理,牢固树立过“紧日子”的思想,厉行节约,勤俭办一切事业,切实把有限的资金用在群众最需要、事业最需要、发展最需要的地方,齐心协力度过暂时的困难。

    四、扎实推进和谐淮北建设

    按照构建社会主义和谐社会的总要求,优化公共资源配置,加快推进以改善民生为重点的社会建设,着力解决人民群众最关心、最直接、最现实的利益问题,共享经济发展的成果。

    大力实施民生工程。进一步加大投入、巩固成果、滚动发展、健全机制,落实好28项民生工程,财政投入总规模超过5亿元。多渠道解决群众住房困难。加强保障性住房建设,新建经济适用房10万平方米,廉租房10万平方米、企业集资建房15万平方米,滨河花园一期下半年交付使用,滨河花园二期实现所有楼栋主体封顶。推进淮北矿业集团棚户区改造,完成仁和小区建设。继续做好就业和再就业工作。实施更加积极的就业政策,千方百计增加就业岗位。继续为下岗失业人员提供岗位补贴、社保补贴、税费减免等优惠政策,鼓励和支持企业更多地吸纳、安置城乡劳动者就业。坚持以创业带动就业,强化职业技能培训和就业援助,大力开发公益性岗位,完善零就业家庭和困难群众的就业帮扶政策,健全覆盖城乡的公共就业服务体系。统筹做好高校毕业生、退伍转业军人等就业工作。加快构建覆盖城乡居民的社会保障体系。扩大社会保险覆盖面,调整企业退休人员养老金、工伤保险等政策标准,建立新型农村养老保险制度,积极稳妥地解决老失地农民的基本生活保障问题。落实城乡最低生活保障制度,进一步提高救助水平。实施重度残疾人生活救助,加强“农村五保”和其他服务机构建设。继续推进济困助学工作。健全学生资助制度,解决好经济困难家庭、进城务工人员子女就学难问题,对城市低保家庭贫困生免费提供教科书,做好普通高校经济困难家庭学生生源地贷款工作。完善城乡医疗救助制度。制定并落实城乡特困群体参加新农村合作医疗、城镇居民医疗保险补助办法和政府购买公共卫生服务实施意见,加快乡镇卫生院、村卫生室和社区卫生服务机构标准化建设。着力改善农村群众的生产生活条件。投资4000万元,再解决10万农村人口安全饮水问题。建设农村公路160公里,基本实现水泥路村村通。加快实施文化资源信息共享、广播电视村村通、乡镇综合文化站、农民体育健身、农家书屋等文化惠民工程,不断丰富农民文化生活。

    积极发展各项社会事业。实施科技兴市和知识产权战略,推动创新型淮北建设。组织实施自主创新重大项目,加快推进科技成果的孵化和转化工作。加强农业技术推广体系建设,完善畜牧防疫体系。发挥专利技术展示交易中心的功能和作用,促进更多的专利技术在淮北转化。全面实施素质教育,继续推进教育均衡发展,建设5 所农村寄宿制初中,完成翠峰路小学、长山南路小学标准化建设和梅苑学校初中综合楼建设,全面实施实验高级中学新校区建设。将教师补充纳入年度常规工作,继续开展教师到薄弱学校支教工作。加强职业教育,积极申办安徽理工职业学院,启动高等教育和职业教育园区建设,支持淮北煤师院新校区建设和更名工作,发展和规范社会力量办学。举办全市首届职业教育技能比武大赛。组织开展好建国60周年庆祝活动;积极推进文化体制改革,不断加强农村和城市社区文化建设,抓好文化、出版物市场管理,加快新图书馆、新广电中心和文化艺术中心建设工作,办好第五届中国大运河文化节和大运河申遗高峰论坛。大力开展全民健身活动,积极备战省十二届运动会,加强市体育中心配套设施建设,加快体校建设,筹建水上运动中心。深入开展爱国卫生运动,深化医疗卫生体制改革,健全疾病预防控制体系, 搞好城乡医疗卫生服务,积极筹建市妇幼保健院二期工程,开工建设市中医院病房大楼,建成市人民医院新病房大楼。加强人口和计划生育工作,完善计划生育工作利益导向机制和“村为主”工作机制,落实计划生育家庭各项奖励扶持政策,加大出生人口性别比偏高综合治理力度,逐步提高基层计生专干的生活待遇。加强国土资源管理工作,大力推进土地整理复垦开发,提高土地节约集约利用水平,进一步加强矿产资源的保护和开发利用。积极推进机关节能工作。加快污染治理基础设施建设,加强重点行业节能减排和农村环境整治,严格环保执法。强化价格监管。继续抓好外事、侨务和对台事务。做好编制、档案、气象、防震减灾、接待、新闻出版等工作。重视和支持妇女、老龄和残疾人事业,加强未成年人保护工作。积极发展社会福利事业和慈善事业。进一步做好民族宗教工作。认真做好第二次全国经济普查工作。加强国防教育,积极支持驻淮部队建设,做好国防动员、民兵预备役和人民防空工作,提升双拥优抚安置工作水平,巩固军政军民团结。

    维护社会大局稳定。进一步加强人民调解工作,拓展和规范法律服务,将“五五”普法向纵深推进。大力推进平安淮北建设,加强基层基础工作,构建社会治安综合防控体系,严厉打击刑事和经济犯罪。建立健全突发性事件预警预防体系,抓好对重大自然灾害等突发事件的监测和应急处置工作。加强和改进信访工作,密切关注信访群众诉求和倾向性、苗头性问题,不断完善社会矛盾纠纷排查调处机制,解决好土地征用、房屋拆迁、涉法涉诉等领域中侵害群众合法权益的问题。加大安全生产监管力度,建立健全安全事故举报制度,突出抓好矿山、消防、交通、食品药品安全、电力设施及电能保护等重点领域的安全监管,杜绝重特大事故发生,大力营造平安和谐的发展环境。

    五、努力建设人民满意的政府

    面对新挑战,实现新发展,是全市人民赋予政府光荣而神圣的职责。我们将继续加强政府自身建设,着力转变职能,切实提高效能,不断开创淮北发展的新局面。

    勤学为务,着力提升施政能力。深入开展学习实践科学发展观活动,用科学发展观武装头脑、指导实践、推动工作,不断增强见微知著的判断能力,果断理性的决策能力,因势利导的掌控能力和务实高效的执行能力。树立战略眼光,着眼全局谋划发展,在顺应宏观调控中抢抓机遇,在适应形势变化中推动发展。树立正确的政绩观,多做打基础、利长远、增后劲的工作,多抓全局性、牵动性、导向性的大事,以通力合作的姿态共克时艰,以多谋善断的智慧展示能力,以时不我待的精神紧抓项目,以科学应对的方法发展自身。破除僵化滞后的思维模式和行为习惯,在完成常规动作基础上,善于拿出自选动作,敢于尝试高难动作,宽容探索中的失误,允许创新中的曲折,善待竞争中的失利,充分激发创造活力。

    公正为上,着力推进依法行政。坚持把依法行政贯穿政府决策、执行、监督的全过程和政府管理的各个环节,努力创造公平竞争的市场环境、稳定透明的政策环境、规范健全的法制环境。完善重大决策程序,畅通社情民意反映机制,健全重大事项社会公示、听证和专家咨询制度,进一步提高政府决策的透明度和公众参与度。加强行政执法监督,严格执行优惠卡制度,认真落实行政问责制、行政执法责任制和过错责任追究制,完善行政复议委员会制度,做好行政复议和行政应诉工作,促进严格执法、公正执法、文明执法。依法接受市人大及其常委会的法律监督,主动接受市政协的民主监督,认真办理人大代表议案、建议和政协委员提案。建立政务公开长效机制,完善政府新闻发言人和政府信息公开制度,充分保障人民群众的知情权、参与权、表达权、监督权。加强政府诚信建设,加大信用监督和失信惩戒力度,做到立言为信、立行必果。

    服务为本,着力提高行政效能。坚持以人为本,全面加强服务型政府建设,始终不渝地实现好、维护好、发展好人民群众的根本利益。适时推进政府机构改革,实施政务流程再造,解决部门之间职能交叉重叠、责任不清和事权分离问题;理顺市与县区政府的职责权限,实现社会管理重心下移。进一步加强全市公务办公网建设,推动市县区政府之间、行政服务中心与窗口机关之间网络互联互通。继续办好“市长热线”和“政风行风热线”,深入开展群众评议机关活动。积极推行工作推进项目化、项目落实责任化,加强目标管理,加大督查力度。多深入实际、多掌握实情、多研究实招,少开会、开短会,少行文、行短文,少评比、评实效。市政府将带头精减会议、精减文件,严格控制各类检查、评比,精简从简、务实有效地举办会展和节庆活动。

    廉洁为要,着力加强廉政建设。坚持立党为公、执政为民,进一步加强作风建设,不断优化政治生态。大力弘扬实干精神,支持干事者、开拓者,反对形式主义、官僚主义,反对弄虚作假和奢侈浪费。认真落实党风廉政建设责任制,建立健全教育、制度、监督并重的惩治和预防腐败体系,用制度管权、按制度办事、靠制度管人。认真履行“一岗双责”,落实述职述廉制度和个人重大事项报告制度。加强经济责任审计,规范工程建设招投标、土地和矿产资源出让、政府采购等公共资源管理权力运行。积极开展专项治理和监督检查,强化对社保基金、住房公积金的监管,重点解决群众反映强烈的突出问题。强化民主监督,发挥舆论监督作用,提高拒腐防变能力。

    各位代表!抓住新机遇就能实现新发展,战胜新挑战才能铸就新辉煌。政府工作的出发点和落脚点,就是为人民服务、让人民满意。在新的一年里,我们将在市委的坚强领导下,在市人大、市政协的监督支持下,紧紧团结和依靠全市人民,谋发展之势,鼓实干之劲,聚和谐之力,以科学发展的新成效,向建国六十周年献礼!
